# Variations in GP–patient communication by ethnicity, age, and gender: evidence from a national primary care patient survey

**DOI:** 10.3399/bjgp15X687637

**Published:** 2015-11-06

**Authors:** Jenni Burt, Cathy Lloyd, John Campbell, Martin Roland, Gary Abel

**Affiliations:** Primary Care Unit, Department of Public Health and Primary Care, University of Cambridge School of Clinical Medicine, Cambridge.; Faculty of Health and Social Care, The Open University, Milton Keynes.; University of Exeter Collaboration for Academic Primary Care, University of Exeter Medical School, Exeter.; Primary Care Unit, Department of Public Health and Primary Care, University of Cambridge School of Clinical Medicine, Cambridge.; Primary Care Unit, Department of Public Health and Primary Care, University of Cambridge School of Clinical Medicine, Cambridge.

**Keywords:** communication, healthcare disparities, minority groups, physician–patient relations, primary health care

## Abstract

**Background:**

Doctor–patient communication is a key driver of overall satisfaction with primary care. Patients from minority ethnic backgrounds consistently report more negative experiences of doctor–patient communication. However, it is currently unknown whether these ethnic differences are concentrated in one gender or in particular age groups.

**Aim:**

To determine how reported GP–patient communication varies between patients from different ethnic groups, stratified by age and gender.

**Design and setting:**

Analysis of data from the English GP Patient Survey from 2012–2013 and 2013–2014, including 1 599 801 responders.

**Method:**

A composite score was created for doctor–patient communication from five survey items concerned with interpersonal aspects of care. Mixed-effect linear regression models were used to estimate age- and gender-specific differences between white British patients and patients of the same age and gender from each other ethnic group.

**Results:**

There was strong evidence (*P*<0.001 for age by gender by ethnicity three-way interaction term) that the effect of ethnicity on reported GP–patient communication varied by both age and gender. The difference in scores between white British and other responders on doctor–patient communication items was largest for older, female Pakistani and Bangladeshi responders, and for younger responders who described their ethnicity as ‘Any other white’.

**Conclusion:**

The identification of groups with particularly marked differences in experience of GP–patient communication — older, female, Asian patients and younger ‘Any other white’ patients — underlines the need for a renewed focus on quality of care for these groups.

## INTRODUCTION

Systematic variations in experience of health care in relation to ethnicity, age, gender, health, and socioeconomic status are well documented.^1^–^6^ In 2014, NHS England reiterated concerns about variations in the quality of primary care for disadvantaged groups, reminding us that ‘People have a right to high quality services, irrespective of who they are, their social status, where they live, or what needs they have’.^7^

The need for action on variations in care is supported by responses to the English GP Patient Survey (GPPS). Analyses based on the 2009 GPPS found that minority ethnic patients (particularly those from South Asian and Chinese backgrounds), patients with poor self-rated health, and younger patients reported more negative experiences of care.^8^ A particular concern is variation in doctor–patient communication, a major driver of overall satisfaction with care.^9^

In the 2009 GPPS analysis, half of the overall difference between South Asian and white patients in reported doctor–patient communication was attributed to the concentration of South Asian patients in practices who did less well overall, receiving lower scores from all patients registered with them, including white British patients. However, the remaining difference reflected less positive reported experience from minority ethnic groups compared with white British counterparts in the same practices.^8^

To consider what actions are required to ensure high-quality care for all, first a more nuanced understanding is required of how patient characteristics might interact with one another in evaluations of care. For example, it would be useful to know whether reports of poorer GP–patient communication are consistent across responders within a particular ethnic group, or whether there are variations according to age. It would also be helpful to know where the largest gaps in experience lie. Interactions between age and ethnicity have been identified for patient reports of the number of GP consultations that take place before hospital referral for cancer.^10^ To explore whether such interactions exist for other aspects of patient experience, 2012–2013 and 2013–2014 GPPS data were analysed to determine how reported GP–patient communication varies between patients from different ethnic groups by age and gender.

## METHOD

The GPPS is sent annually to around 2.7 million patients in England who are registered with a general practice for at least 6 months. Full details of the survey and methodology are published elsewhere.^11^–^13^ To increase the number of responses for small ethnic groups in the analysis, data were combined from 2 years of the survey (2012–2013 and 2013–2014). As no patient receives the survey in two consecutive years, there is no risk of double counting.

**How this fits in**

Patients from minority ethnic groups report more negative experiences of primary care, including doctor–patient communication, than their white British counterparts. This analysis of GP Patient Survey data reveals that the effect of ethnicity on reported GP–patient communication varies by age and gender. Older, female, Asian patients and younger ‘Any other white’ patients have particularly marked negative experiences of GP–patient communication compared with white British patients. The practice of patient-centred medicine, awareness of the challenges in cross-cultural consultations, and system-level initiatives to better support disadvantaged groups are all important in addressing these inequalities of care.

A measure of reported GP–patient communication was constructed from five sub-items following the stem ‘Last time you saw or spoke to a GP from your GP surgery, how good was that GP at each of the following?’. These were: ‘Giving you enough time’, ‘Listening to you’, ‘Explaining tests and treatments’, ‘Involving you in decisions about your care’, and ‘Treating you with care and concern’. Each had a 5-point Likert scale response from ‘Very good’ to ‘Very poor’, as well as ‘Doesn’t apply’ (which was classified as an uninformative response option). A composite score was created for all responders who provided three or more informative responses by linear rescaling of the responses between 0 and 100, and taking the mean of all sub-items answered. Patient-reported age group, gender, and ethnicity were taken directly from survey responses. Further, health-related quality of life was measured using responses to five questions that make up the EuroQol EQ-5D-3L descriptive system.^14^,^15^ The Index of Multiple Deprivation, an area-based measure of socioeconomic status based on the patient’s residential postcode, was also available.^16^ For analysis, this was split into five groups based on national quintiles.

A mixed-effect linear regression model was used with the GP–patient communication score as the outcome. The model included age, gender, ethnicity, EQ-5D, and deprivation as fixed effects, and a random effect for practice (to account for the fact that certain patient groups cluster in practices that may perform better or worse overall). All possible two-way interactions between age, gender, and ethnicity, as well as the three-way interaction between them were included in the model to allow the effect of ethnicity to vary between different age and gender groups. Wald tests of the interaction terms were used to assess evidence supporting this variation. The models were then used to estimate age-and gender-specific differences between white British patients and patients of the same age and gender from each of the other ethnic groups. All analyses were carried out using Stata (version 13.1).

## RESULTS

**Figure 1.**
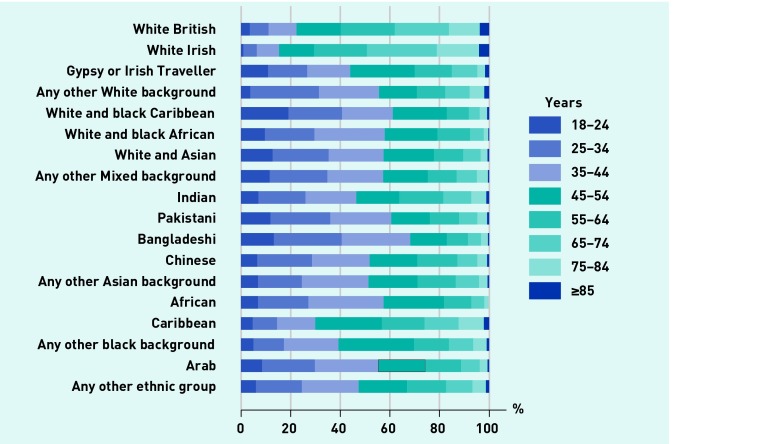
***Age composition of responders according to self-reported ethnicity.***

Across 2012–2013 and 2013–2014, GPPS received 1 874 589 responses, an overall response rate of 35%. Of these, 1 599 801 (85%) had complete data for all items in the analysis. The numbers of responders in each ethnicity group are shown in Table 1. The largest group of responders were white British (*n* = 1 323 621, 82.7%): there were at least 1800 responders in all but one group. Figure 1 shows the age composition of each group. White British and white Irish responders tended to be older than those from other ethnic groups, and are dominated by those aged ≥55 years. For nearly all other ethnicities most responders were aged ≤45 years. Consequently, there were very few responses in the oldest age groups for a number of ethnicities (Table 1).

**Table 1.** Ethnicity make-up of sample for all ages and those aged ≥85 years

**Ethnicity**		**All ages**		**≥85 years**	

		***n***	**%**	***n***	**%**
	
White	British	1 323 621	82.7	49 891	93.1
	
	Irish	16 330	1.0	662	1.2
	
	Gypsy or Irish Traveller	401	0.0	6	0.0
	
	Any other white	71 105	4.4	1386	2.6
	
Mixed/multiple ethnic groups	White and black Caribbean	3413	0.2	26	0.1
	
	White and black African	1865	0.1	4	0.0
	
	White and Asian	3171	0.2	18	0.0
	
	Any other mixed	3340	0.2	15	0.0
	
Asian/Asian British	Indian	38 705	2.4	425	0.8
	
	Pakistani	20 729	1.3	143	0.3
	
	Bangladeshi	6699	0.4	23	0.0
	
	Chinese	7986	0.5	66	0.1
	
	Any other Asian	19 812	1.2	105	0.2
	
Black/African/Caribbean/Black British	African	21 131	1.3	24	0.0
	
	Caribbean	13 715	0.9	275	0.5
	
	Any other black	6061	0.4	52	0.1
	
Other ethnic group	Arab	2786	0.2	16	0.0
	
	Other	38 931	2.4	458	0.9
	
**Total**		**1 599 801**	**100.0**	**53 595**	**100.0**

**Figure 2.**
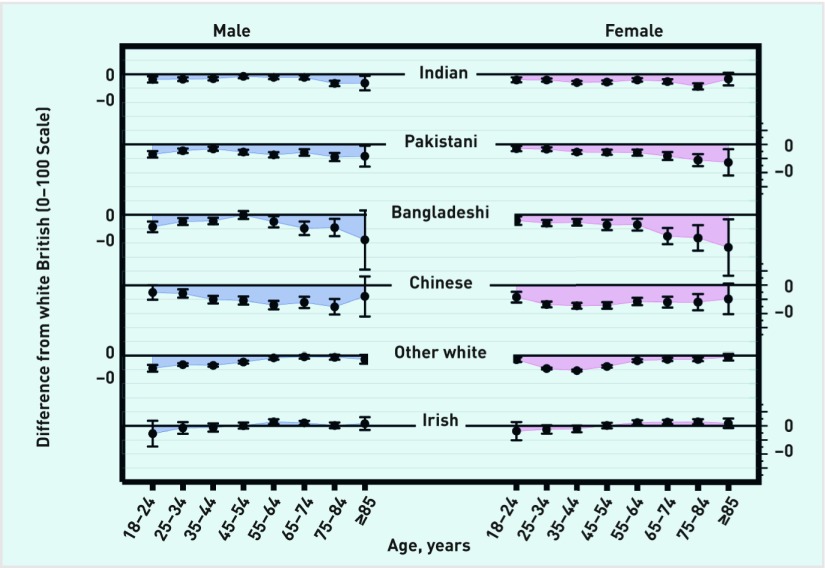
***Age and gender-specific differences, with 95% confidence intervals, in reported GP–patient communication scores (0–100 scale) between white British patients and responders in Asian and white ethnic groups.***

From the regression model (adjusting for deprivation, EQ-5D, and practice), there was strong evidence (*P*<0.001 for age by gender by ethnicity three-way interaction term) that the effect of ethnicity on reported GP–patient communication varied by both age and gender (further details are available from the authors on request). This variation is illustrated in Figure 2, which shows the age- and gender-specific adjusted differences between white British responders and responders of the same age and gender from all Asian sub-groups and white (non-British) ethnic groups: negative differences indicate responders reported worse experience than their white British counterparts (that is, of the same age and gender). The Asian and white (non-British) responses are highlighted as the ethnic groups where the largest differences are seen.

Differences in reported experience of GP–patient communication between Asian groups and the white British group were largest for older responders (≥55 years). This differential effect of ethnicity was particularly marked in Bangladeshi responders, and for females (Figure 2). For example, the difference in reported experience scores between a white British 75–84-year-old female and a Bangladeshi female of the same age was −8.23 points on a 0–100 scale (95% confidence interval [CI] = −12.76 to −3.69). However, for Indian, Pakistani, and Bangladeshi groups, the differences in younger age groups compared with white British responders were fairly small. For example, the difference in reported experience score between a white British 35–44-year-old female and a Pakistani female of the same age was −2.72 points (95% CI = −3.42 to −2.02) (Figure 2). For Chinese responders, substantial negative differences compared with white British counterparts were seen across all age groups.

In contrast to Asian responders, for ‘Any other white’ responders, the ethnic disparities in reported GP–patient communication were largest for younger responders (<55 years). For example, the difference in reported experience score between a white British 35–44-year-old female and an ‘Any other white’ female of the same age was −5.30 points (95% CI = −5.66 to −4.95). Again, these differences were larger for females than males. For Irish compared with white British responders there were few disparities; small negative differences for younger (<45 years) responders and small positive differences for older (≥45 years) responders. For ethnic groups not shown in Figure 2 (further details on age and gender-specific differences are available from the authors on request), few differences were found in reported experience at all ages for African, Caribbean, and other black responders. The ability to detect differences for mixed ethnic groups was limited: CIs are generally large, reflecting the smaller sample sizes. However, there were more substantial (and statistically significant) negative differences for other Asian females (at all ages), and for white and Asian females (particularly at older ages).

## DISCUSSION

### Summary

This analysis of GPPS data has shown that the effect of ethnicity on reported GP-communication varies by age and gender. In comparison with white British responders of the same age and gender, poorer experience scores for GP–patient communication are particularly marked in older, female, Asian patients, and in younger ‘Any other white’ patients.

### Strengths and limitations

GPPS data are derived from a large, randomly selected sample designed to be representative of patients registered with a practice in England.^11^,^12^ While inclusion of over 1.5 million patients enables precise measurements of overall experience, the ability to derive precise estimates in relation to age is limited in the smallest ethnic groups (such as Arab, and Gypsy and Irish Travellers).

Response rates to the GPPS are low: for the years analysed these were 35% and 34%, respectively. Recent syntheses suggest response rates are not a strong indicator of non-response bias in surveys that use probability sampling.^17^ If present, non-response bias is more likely to affect absolute scores than the relative scores presented here. For non-response bias to be driving the findings, the association between experience and the likelihood of responding to a survey would need to be differential between ethnic groups, favouring responses from patients from minority ethnic backgrounds with negative experiences but not white British patients with negative experiences. This seems unlikely. Non-response bias is more likely to attenuate differences due to difficulties accessing those with low English language proficiency. While the GPPS is offered in 13 additional languages, in the years analysed only 0.2% of patients completed the questionnaire in a language other than English (most being Polish). If survey responders are more proficient in English, this may underestimate the communication difficulties experienced by certain minority ethnic groups, as those with the greatest communication difficulties will be excluded from the study sample.

Finally, as no objective measure of GP–patient communication exists for these data, the analysis is not able to provide insight into whether reported experience varies as a result of differences in actual experience or differences in reports of experience as a result of variations in expectations or survey response tendencies: for this, experimental approaches are required.^18^

### Comparison with existing literature

Previous analyses have identified variations in patient experience in relation to ethnic group, age, and gender, and have found an interaction between ethnicity and age for cancer referrals.^8^,^10^,^19^ The authors believe this study is the first to consider the interactions between all three factors to explore their impact on reported GP–patient communication. The analyses highlight two groups of particular concern: older, female, Asian patients and younger ‘Any other white’ patients. These groups reflect distinctly different profiles and patterns of migration to the UK: however, patients from these groups may face similar barriers, including poor language proficiency, lack of acculturation, and provider-side discrimination.

Language is only one part of communication, but an important one. Language-discordance occurs when a doctor and patient do not share the same language. The proportion of those who cannot speak English well or at all varies widely between and within ethnic groups: 16.2% of Bangladeshi census responders, 15.2% of Chinese, 12.2% of ‘Any other white’, and 11.1% of Pakistani patients fall into this category.^20^ Older Bangladeshi and Pakistani females may be prevented from acquiring English proficiency through family obligations, or cultural and community expectations.^21^ The ‘Any other white’ group contains a large proportion of Polish-born responders, including a younger, less established population whose employment and social interactions may make it difficult to develop English proficiency.^22^–^25^ A number of studies have suggested that language discordance in clinical encounters may negatively impact on quality of care.^26^–^29^ Challenges in communicating in language-discordant consultations can lead to particularly strong tensions between ‘ideal’ standards of communication and what is ‘good enough’.^30^

Acculturation is concerned with the modification of attitudes or behaviours as people come into contact with a culture other than their own: although its definition and scope are contested, it is frequently used to explain inequalities in health care.^31^ Levels of acculturation may lead to variations in perceptions and expectations of providers and care, and ability to navigate the healthcare system, impacting on reported experience.^32^ Previous analysis of patient experience in US primary care for Hispanic patients found no relationship between acculturation levels and patient reports of provider communication, although there was an association with other aspects of patient experience.^32^ However, the measurement of acculturation through commonly-used language proficiency scales has been criticised for failing to capture its multidimensional nature.^33^ Further, a focus on lack of acculturation as a driver of disparities may mask other causal factors, including poverty, the social construction of ethnic identities, and inequities in treatment.^34^ Nevertheless, the broad concept of acculturation may be a useful reminder that age, gender, and ethnicity groupings could vary in their understanding and navigation of primary care for reasons that are additional to those of language barriers.

Concerns about institutionally-ingrained variations in attitudes to patients on the basis of ethnicity have led to a rise in cultural-competency training.^35^,^36^ These approaches have been criticised for placing emphasis on patient characteristics as the drivers of variations in care, rather than on provider- and system-level factors, including the potential for stereotyping of, or bias towards, particular groups.^37^ However, this analysis shows that any provider-or system-side factors do not occur in reaction to ethnicity alone, but in response to the inter-relationship between ethnicity, gender, and age. It is the combination of these factors which may identify groups with particular needs, such as those patients with the lowest levels of English proficiency. We therefore need to focus not just on differences between groups but also on differences within them, considering how ethnicity, gender, age, and other categories of social identity interact with each other to create different experiences and outcomes: the study of such interactions has been termed intersectionality.^38^

### Implications for research and practice

The identification of those with particularly marked differences in experience of GP–patient communication — older, female, Asian patients and younger ‘Any other white’ patients — underlines the need for a renewed focus on these groups. For practitioners, the acknowledgement that certain patients may experience greater challenges in communicating is an important first step. Likewise, an awareness of the particular difficulties and frustrations encountered on both sides in cross-cultural consultations is important. Empathy, curiosity, and respect are crucial to engaging with the dynamics which can arise from difference.^39^ Caring for diverse patient populations is an immense challenge: drawing on the principles of person-centred medicine is a useful framework through which to approach this task.^40^ For patients, for example those with limited English language proficiency, effective support for communication in the form of professional interpreters is important.^41^ However, system-level as well as patient-targeted initiatives to improve health literacy are also key, yet inevitably require further resources.^42^ Finally, for researchers wishing to identify the drivers of these observed variations in care, further understanding is needed of expectations, reporting, and experiences of care in these groups. The authors are currently undertaking experimental work with white British and Pakistani communities to determine in more detail where the key issues lie.
